# Resilience of Iranian women in natural disasters: a qualitative study

**DOI:** 10.1186/s12889-025-24828-0

**Published:** 2025-10-21

**Authors:** Mozhdeh Zarei, Abbas Ostadtaghizadeh, Ali Ardalan, Kiyoumars Allahbakhshi, Abbas Ebadi, Abbas Rahimi Foroushani

**Affiliations:** 1 Department of Health in Emergencies and Disasters, School of Public Health, Tehran University of Medical Sciences, Tehran, Iran; 2 Deputy of Research and Technology, Kurdistan University of Medical Sciences, Sanandaj, Iran; 3 Nursing Care Research Center, Clinical Sciences Institute, Baqiyatallah University of Medical Sciences, Tehran, Iran; 4 Department of Epidemiology and Biostatistics, School of Public Health, Tehran University of Medical Sciences, Tehran, Iran

**Keywords:** Resilience, Women, Gender, Natural disasters, Iran, Qualitative study

## Abstract

**Background:**

Although natural disasters pose a threat to everyone, they do not affect all members of society equally. It is now recognized that disasters have gendered dimensions, with different effects on men and women. Resilience is a challenging concept that has become a concern for researchers in the field of health during disasters and emergencies. This study was conducted to identify the resilience characteristics of Iranian women in natural disasters.

**Materials and methods:**

This study used a qualitative approach, employing conventional content analysis based on Lindgren’s (2020) method. Purposeful sampling was used to conduct individual interviews with 11 women aged 18 and older who were affected by natural disasters, as well as 10 experts in disaster management and resilience.

**Results:**

The resilience of Iranian women in natural disasters was categorized into four main categories: individual resilience (demographic factors, personality traits, physical and mental aspects, knowledge, skills, and previous experiences), cultural resilience (gender discrimination, norms, religious beliefs and practices), social resilience (social interactions, social support, and community security), and economic resilience (assets and income).

**Conclusion:**

To improve the resilience of women in natural disasters, it is necessary to address gender discrimination in policies and planning, including ensuring women’s equitable access to education, property ownership, employment, and leadership positions. The root causes of discrimination in various cultural, social, political, and economic dimensions must be addressed. We recommend that disaster management policies include specific components to guarantee the protection of women’s legal rights in different communities.

## Introduction

In 2024, the Emergency Events Database (EM-DAT) recorded a total of 393 natural disasters. These events resulted in 16.753 deaths, affected 167.2 million people, and caused $241.95 billion USD in economic damage [1]. With the ever-growing global population, natural disasters have caused significant casualties, severely impacting the well-being and safety of individuals, communities, and nations. Since the mid-1990s, more than 1.5 billion people have been affected by natural disasters in various ways [2].

It has been established that disasters are gendered events, and women and girls bear a greater burden from disasters than men do and suffer from long-term effects such as increased violence and insecurity [3]. While disaster management planning and efforts should be inclusive and focus on gender issues, this has not been adequately addressed in many disaster risk reduction policies [4]. The “Gender and Natural Disaster Risk Reduction” conference stated that women are a key point for addressing disaster risks and building resilience [5]. In 2012, the United Nations Commission recognized the vital role of women in reducing the impacts of natural disasters, responding to them, and recovering from them. The report “Gender Equality and Empowerment of Women in Natural Disasters” also emphasized the significant role of women in the recovery phase and the adoption of a gender-based approach to disasters. International and national policies must enhance the inclusion of gender perspectives, which are currently considered insufficient within international frameworks such as the Sendai Framework for Disaster Risk Reduction 2015–2030 [6].

The vision for global peace and prosperity outlined in the Sustainable Development Goals (SDGs) cannot be fully realized without addressing resilience [7]. Resilience, considered a core element of sustainable development, is a multifaceted concept that has been defined by researchers across different disciplines. One of the most recent and significant applications of this concept is within the disaster paradigm [8]. Community and group resilience are integral to disaster risk reduction (DRR), with DRR initiatives aimed at understanding, reducing, and preventing risks to build resilient communities and promote sustainable development [9]. These programs strengthen community capacities to respond to disasters, helping to maintain essential functions and accelerate recovery after events, ultimately facilitating sustainable development [10]. Resilience encompasses the capacity of a community to resist, prepare, adapt, change, and recover promptly and effectively while enduring natural hazards [11]. Resilience, as a complex and multidimensional concept, requires thorough examination within cultural and gender contexts to develop effective strategies for its enhancement [12]. Although numerous studies have been conducted on resilience and valuable research has been conducted on women’s resilience, there have been very few studies on women’s resilience in natural disasters globally and in Iran. The studies conducted in Iran are quite limited and have focused on specific groups of women, such as rural women [13], or have solely examined gender during disasters [14].

Given the strong dependence of the concept of resilience on gender and culture [15, 16], conducting qualitative studies with a comprehensive and context-specific approach is essential. Such studies are particularly valuable for documenting the lived experiences, perceptions, and coping strategies of individuals and communities affected by disasters. While quantitative approaches provide important data on resilience indicators, they often overlook the subtle, subjective, and culturally embedded ways in which people perceive and respond to adversity. Qualitative methods, such as in-depth interviews and narrative inquiry, enable researchers to uncover the complexities of disaster experiences, including the social and cultural dimensions that shape resilience. This study was conducted in response to the scarcity of qualitative research in this field and aims to provide deeper insights into the factors influencing the resilience of Iranian women in the face of natural disasters.

## Materials and methods

### Design

This qualitative study employed conventional content analysis to explore and derive meaning from the collected data. The aim of content analysis is to organize and extract meaning from the data and draw realistic conclusions from it [17]. In conventional content analysis, researchers avoid using predetermined categories and instead allow categories to emerge directly from the data [18]. Therefore, data were collected without any prior hypotheses, and the codes, subcategories, and categories were inductively derived.

Iran is a highly vulnerable country where natural disasters, such as floods and earthquakes, are frequent [19]. This study was conducted in Kermanshah, which was affected by a 7.3 magnitude earthquake on November 12, 2017, and in Gilan Province, which was impacted by a major flood in 2019. These two provinces were selected because of the recent occurrence of these disasters and easier access to affected individuals.

### Participants

The interviews were conducted at two levels in Iran: with key informants—individuals with scientific and practical knowledge in the area of health during emergencies and disasters—and with women over the age of 18 who had been affected by one of these natural disasters.

Participants in this study were selected through purposive sampling to identify individuals capable of providing the most relevant and in-depth information regarding the resilience of Iranian women in the context of natural disasters. This sampling method, one of the most commonly employed in qualitative research, is based on the researcher’s judgment and understanding of the target population. Its primary objective is to achieve a deep and comprehensive understanding of the phenomenon under investigation, rather than to generalize the findings to the entire population [20]. Accordingly, interviews in this study were conducted at two levels:

- First Group
  The first group of participants consisted of women who had experienced at least one natural disaster, such as a flood or earthquake, and were willing to participate in the interviews. Since this study specifically focused on the resilience of women and girls, participants under 18 years of age were excluded [21]. These interviews were conducted face-to-face, either at the participants’ homes or at local health service centers, depending on their preference.
- Second Group
  The second group comprised experts with a minimum of five years of professional experience related to disaster management, resilience, and women’s issues. Given the multidimensional nature of resilience, individuals with relevant expertise and experience were purposively invited from various organizations, including universities, research institutes, women’s charitable organizations, the Imam Khomeini Relief Committee, the Red Crescent Society, and the Welfare Organization. Interviews with these experts were conducted either by telephone or at their workplaces, according to their preference.

Despite prior arrangements, two participants could not attend the interviews because of work commitments. In total, 21 eligible participants were included in the study (see Table 1).

**Table 1:** Information of the participants in the qualitative study of Iranian women’s resilience in natural disasters

Variable	Variable description
Experience	**From women with disaster experience**: 11 Cases (52%)
	• 5 Cases (45.50%) have experienced floods
	• 6 Cases (54.50%) have experienced earthquakes
	**From Experts**: 10 Cases (48%)
	• 4 Cases (40%) academic members specializing in health in emergencies and disasters, crisis management, and social sciences
	• 1 Case (10%) midwife
	• 1 Case (10%) rural health worker
	• 2 Cases (20%) emergency responders
	• 2 Cases (20%) psychologists
Gender	Male: 4 Cases (19%) Only from Experts
	Female: 17 Cases (81%) 6 from experts and 11 from women with disaster experience
Age	2 Cases (9.5%) 18-30Y
	5 Cases (23.8%) 31-40Y
	7 Cases (33.4%) 41-50Y
	6 Cases (28.5%) 51-60Y
	Over 60 Years Old: 1 Case (4.8%): Only a woman with disaster experience
Education	Illiterate: 1 Case (4.8%)-only women with disaster experience
	Primary education: 1 Case (4.8%)-only women with disaster experience
	Middle school and high school diplomas: 4 Cases (19%)-only women with disaster experience
	Associate degrees and bachelor’s degrees: 3 Cases (14.3%)
	Master’s degrees and higher: 12 Cases (57.1%)−2 Cases from women with disaster experience, 10 Cases from experts
Interview Methods	Face-to-face: 14 Cases (66.7%)
	By phone: 7 Cases (33.3%)

### Data collection

The data for this study were collected through semi structured individual interviews, a method that is well suited for qualitative research because of its depth and flexibility [22].

The primary focus of the questions was on factors related to the resilience of Iranian women during natural disasters. After 18 interviews were conducted over a 10-month period (May 2022-December 2022), no new information emerged, and to ensure data saturation, two additional interviews were conducted. Each identified concept was discussed during the interviews until saturation was reached, meaning that no new information or insights were generated. In qualitative research, data saturation refers to the point at which additional data collection no longer produces new information, patterns, or themes, indicating that the analysis has comprehensively covered all relevant aspects of the study. This concept serves as a criterion for concluding sampling and data collection, ensuring that the analysis is thorough and trustworthy while preventing unnecessary repetition of interviews [23]. Following the 5.9 magnitude earthquake in Khoy on January 28, 2023, one more interview was conducted in April 2023, bringing the total number of interviews to 21.

The in-person interviews lasted between 25 and 75 min, whereas the phone interviews ranged from 21 to 70 min. In cases where ambiguities were noted, a second interview was conducted for clarification (this occurred in one case).

Prior to each interview, to build trust and provide a safe and comfortable environment, the purpose and significance of the study were thoroughly explained to the participants. This explanation included a detailed account of the research objectives, the intended use of the data, assurances of confidentiality and privacy, and an emphasis on the crucial role of their participation in contributing valuable insights. Additionally, participants were given the opportunity to ask any questions or express concerns to ensure informed consent was obtained and to facilitate effective collaboration.

The semi-structured interview guide was developed based on the research objectives and a review of relevant literature. The questions (see Table 2) were designed to be open-ended and flexible, and two pilot interviews were conducted to enable a deeper exploration of the topics. The guide was subsequently reviewed and refined by the research team to ensure its clarity and comprehensiveness. This approach facilitated the collection of accurate and reliable data [24].

The interviews were designed based on the participants’ experiences and contexts. Each interview session followed a structured interview guide comprising a predetermined set of questions. Additionally, supplementary probing questions were posed based on the participants’ initial responses and the interview guide. To enhance the depth of the data, exploratory questions such as “What do you mean by that?” or “Could you please elaborate with an example?” were employed. The sequence of questions varied according to the flow of the interview and the participants’ answers. The main interview questions included the following:

**Table 2:** Core interview questions for identifying the components of resilience among Iranian women in natural disasters

Question Number	Interview Question
1	Please describe your experience during the occurrence of the flood/earthquake. What actions did you take at that time?
2	Following the disaster, what obstacles did you encounter in returning to normal life, and how did you overcome them?
3	What skills or personal characteristics helped you maintain your performance during the crisis?
4	Based on your experience during the flood/earthquake, what types of women were less affected by these events?
5	Based on your experiences, what kind of women are able to maintain their performance during natural disasters and return to their normal functioning correctly and in a timely manner?
6	The final question in all interviews was: Is there anything you would like to add to what has already been discussed?

### Data analysis

In this study, data analysis was performed using the conventional content analysis approach based on Lindgren’s approach, version 2020 [25]. The Lindgren (2020) content analysis approach was selected because it is recognized as a rigorous and credible method for qualitative data analysis, enabling the inductive extraction of categories and themes without reliance on predetermined frameworks. By providing a systematic and transparent process for identifying and organizing deeper meanings within textual data, this method is particularly well-suited for qualitative research focused on exploring complex and context-dependent phenomena. Consequently, it enhances both the credibility and the richness of the study’s findings [25].

The audio recordings were transcribed verbatim immediately after each interview. The coding process involved extracting meaning units and then assigning codes by identifying specific parts of the text that contained key concepts or thoughts. Different codes were compared and categorized on the basis of their similarities and differences. The codes were then analyzed and organized into main categories. Recoding was performed if necessary. To ensure a deep understanding of the concepts and to avoid superficial and mechanical coding, the coding and categorization of the concepts were performed manually via paper and pencil, following these steps:

1. Reading: Carefully reading all the interviews to gain a general understanding of the content.
2. Identifying Meaning Units: Recognizing the meaning units within the text.
3. Condensing and Coding: Extracting condensed meaning units and assigning codes.
4. Forming Categories: Continuously comparing codes to form categories and subcategories on the basis of similarities and differences.

All interviews were conducted by MZ, with some sessions held in Kurdish, her native language, and the remainder conducted in Persian, in which she possesses full proficiency. The researchers acknowledge that MZ’s experiences as a woman and mother, along with her shared language and many sociocultural beliefs and practices, may have influenced the research process. However, the interviewer had no personal experience with natural disasters. On the other hand, her background in women’s empowerment programs, years of interaction with women through her midwifery profession, education in health in emergencies and disasters, and training in interpersonal communication skills are strengths that enhanced her ability to collect data effectively.

### Trustworthiness

To ensure trustworthiness, four strategies have been proposed. Schwandt et al. [26]ensured the credibility of the data through member checks, peer review, ongoing comparisons, and triangulation. Additionally, sections of all the interviews were transcribed, and the initial and final codes and categories were verified by a qualitative research specialist. Constant comparison was conducted through repeated returns to the data during the analysis phase, aiding in the formation of categories and subcategories. Two external reviewers with qualitative research skills reviewed the quality of the interviews, coding, and categorizations to achieve consensus. The confirmability of the data was ensured through the use of bracketing, note-taking, and the neutrality of the research team. A detailed description of the entire study process is provided. Transferability for the first group of participants (women) was ensured through maximum sample diversity in demographic characteristics such as age, employment, socioeconomic status, place of residence, and education, as well as concerning factors such as marital status, pregnancy, and breastfeeding. Transferability for the second group of participants (professionals) was considered through maximum sample diversity in terms of experience, education, workplace, and type of responsibility by consulting relevant charities for women, the Imam Khomeini Relief Committee, the Red Crescent, the Welfare Organization, health specialists in emergencies and disasters, and experts in resilience, social sciences, and psychology. For research dependency, a detailed description of the entire study is provided along with a comprehensive descriptive report on collection, classification, and analysis.

## Results

At the beginning of the analysis, 487 codes were obtained. The extracted codes were categorized on the basis of their similarities and differences. The most frequently occurring codes in the interviews included educational attainment (21 times), gender discrimination (20 times), employment (18 times), and economic status (16 times).

**Fig. 1:**
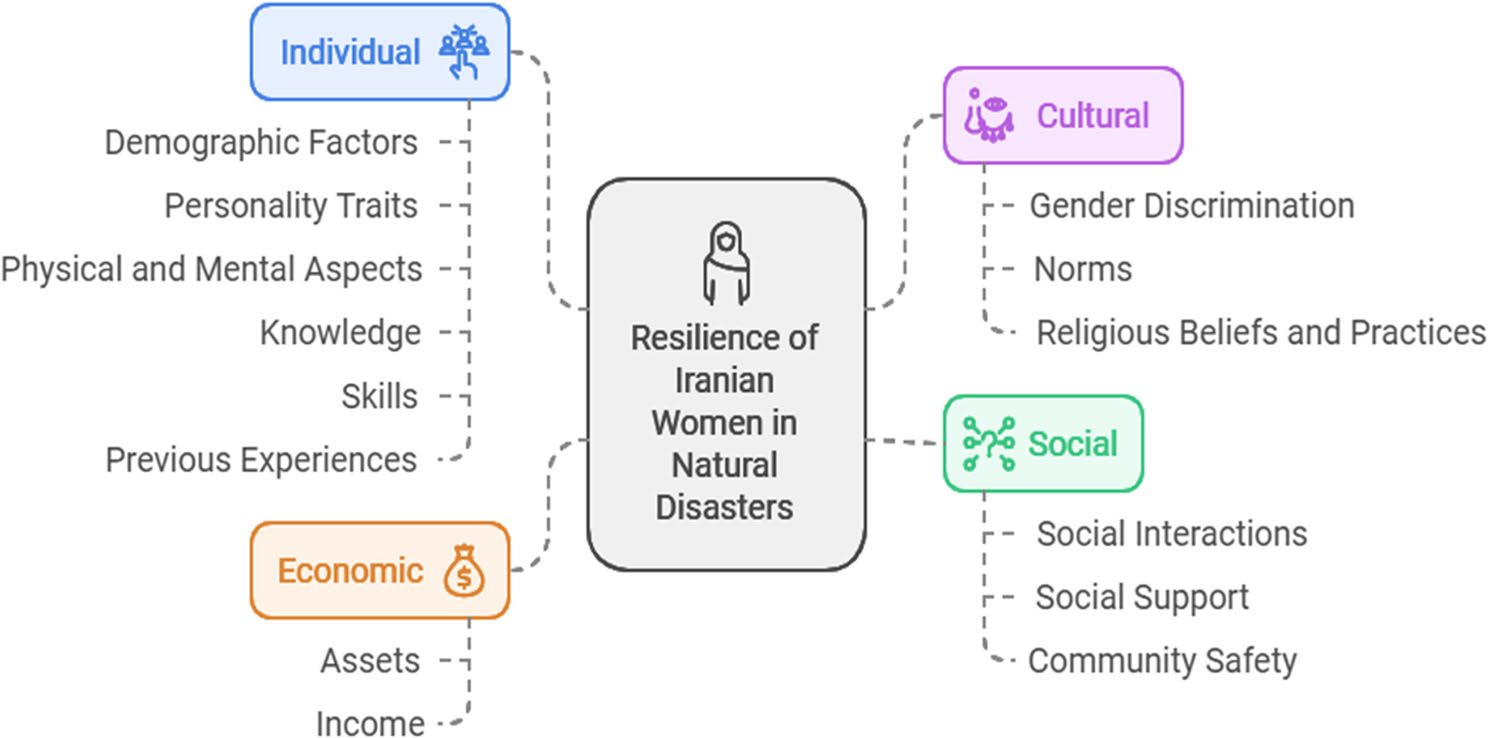
Categories and subcategories emerging from the findings

After the data were analyzed, four main categories and 14 subcategories related to the resilience of Iranian women in natural disasters were identified, including individual factors; such as demographic factors; personality traits; physical and psychological aspects; knowledge, skills, and previous experiences; cultural factors, such as gender discrimination; norms; beliefs; religious practices; social factors; social participation and interaction; social support; community safety; and economic factors, such as financial capability and income (see Fig. 1).

## Individual factors

Key individual factors enhancing Iranian women’s resilience in natural disasters included: (1) Demographic Factors, (2) personality traits, (3) physical and psychological health, (4) knowledge, (5) skills, and (6) previous experiences.

### Demographic factors

The participants indicated that factors such as age, ethnicity, education, and being the head of the household influence women’s resilience during natural disasters. They highlighted that the presence of multiple demographic factors together, such as being elderly or less educated, can severely weaken resilience.

With respect to being the head of the household, most participants believed that this role negatively affects resilience, making it more difficult for women to cope with disasters. However, one participant (Participant 3) provided a contrasting view, suggesting that being the head of the household strengthens women’s capabilities, allowing them to show greater resilience in disaster situations:

- Participant 3: “*Widowed women managed their situations much better. For example*,* our neighbor*,* who raised four children alone without her husband*,* was exceptionally strong during the earthquake because she had always handled all household responsibilities on her own and knew how to manage them during crises*”—P3 from women.
- Experts: “*Women who are heads of their families face greater pressure and responsibilities even under normal circumstances*,* which naturally makes it much harder for them during disasters. When a husband is present*,* responsibilities are shared*,* but in their absence*,* the entire burden falls on one person. A woman who is already under the strain of family responsibilities before an earthquake or flood will find her problems and duties multiplying during the disaster*”—P16.

### Personality traits

Participants emphasized that intrinsic personality characteristics, especially optimism and humor, served as critical adaptive resources when confronting disaster conditions.

- Expert’s view: “*Certain traits*,* like having a sense of humor—something not every woman or individual possesses—are advantages. Generally*,* a humorous woman is more resilient because she perceives that life is not always so serious*,* and this outlook enhances her resilience*.” —P18.
- Participant’s view: “*Women who were delicate and*,* as we say*,* overly sensitive*,* could not endure the hardships. For example*,* my sister-in-law*,* who had been married for just two years— (pauses*,* with emotion)—was forced to move into my father’s house and live in a tent in the yard after the earthquake. She couldn’t handle the situation due to her very sensitive nature*,* and their life fell apart*,* leading to their separation*.” —P2 from women.

### Physical and emotional aspects

The participants indicated that both physical and psychological conditions significantly affect the resilience of women during natural disasters. For example, women who are ill, breastfeeding, pregnant, or menstruating are particularly vulnerable, which reduces their resilience in such situations.

- Participant’s experience: “*I got my period right when the earthquake struck*,* and I did not have access to sanitary products. I was incredibly frustrated. You truly don’t understand how difficult it is to deal with an earthquake*,* with everything in chaos*,* and at the same time be on your period without any hygiene products*,* unless you’ve been in that situation*.” — P7 from Women.
- Another participant’s experience: “*I was extremely worried about my child. I didn’t know if he had survived*,* with all the fear and terror I was feeling. My friend*,* who was also pregnant*,* was in a similar state. For those who were pregnant*,* it was even more challenging because we were anxious about the baby inside us. We were scared*,* and at the time*,* there was no access to medical care or ultrasounds. I went for an ultrasound a week later*,* and I was so terrified that I felt like I might die of fear until the doctor told me that the baby’s heart was healthy*.” — P5 from Women.

### Knowledge

The participants identified knowledge and awareness of the necessary actions during disasters as crucial factors in women’s resilience. They emphasized that, despite the importance of this issue, proper and adequate training is not provided consistently, neither in schools nor through media.

- Expert opinion: “*In all disasters*,* it has been observed that women who possess the necessary knowledge and awareness—such as knowing what to do when a flood warning is issued—are significantly less affected by disasters. Unfortunately*,* the importance of education becomes evident only when disasters strike*,* and by then*,* it is often too late. Prior to such events*,* training is scattered*,* infrequent*,* insufficient*,* and lacks coordination*.” — P13.

*“When the earthquake struck*,* I didn’t know whether to take cover under furniture or attempt to escape. I later learned that our neighbor immediately guided the children to shelter under a doorframe*,* saving them all. Had we received even basic training*,* my mother might have survived the collapsing debris”* — P2 from Women.

### Skills

The participants indicated that physical preparedness and skills such as swimming and running significantly enhance women’s resilience during natural disasters. However, most participants noted that societal norms often limit women’s opportunities to acquire such skills.

- Participant account: “*Women here rarely go to gyms or pools because they are usually busy with household chores and childcare. Even if they have the time*,* they often do not have permission. I engage in physical fitness training and were in good shape. During the earthquake*,* I managed to get to the yard before anyone else. In a situation where no one expected it*,* I went back and rescued my nephew*,* who was still asleep*,* from under a collapsing wall. My family and I were astonished by my ability to react so quickly*.” — P9 from Women.

*As a relief worker*,* I have repeatedly observed that women who possess physical skills such as swimming or running are able to respond more effectively during disasters. Unfortunately*,* due to prevailing social norms in society*,* many women are deprived of the opportunity to learn these vital skills.* — P12 from experts.

### Previous experiences

Participants reported divergent perspectives regarding prior disaster exposure: while some described enhanced response capabilities during subsequent crises, others emphasized that repeated traumatic experiences led to psychological depletion.

- Participant account: “*Women who have experienced severe and repeated traumas may struggle*,* but those who have faced previous problems and stresses without being overwhelmed tend to be more resilient during disasters. Experiencing hardships is similar to vaccination; when you encounter and solve a problem*,* it stays in your memory*,* allowing you to know how to act in similar situations in the future*.” — P19 from experts.
- Another participant commented: “*Women who lived through war were more resilient because they experienced harsh conditions with inadequate housing and minimal resources. For example*,* my mother handled the earthquake very well. She would say*,* ‘I’ve seen much worse conditions. An earthquake is nothing I can’t endure. ' She would shrug and smile*,* ‘It is not worse than war.*‘” — P4 from Women.

## Cultural factors

Cultural factors contributing to Iranian women’s resilience in natural disasters comprised three key subcategories: (1) gender discrimination, (2) social norms, and (3) religious beliefs and practices.

### Gender discrimination

Participants emphasized structural barriers that diminished women’s disaster resilience, particularly highlighting restricted decision-making autonomy, unequal access to critical resources, and deeply rooted patriarchal social norms.

An expert explained, “*In some communities*,* women are not allowed to leave the house without their husbands’ permission. During disasters; such as earthquakes*,* these women face even more severe restrictions*,* exacerbating their challenges. For example*,* there was a woman whose husband did not allow her to leave the tent*,* even to seek psychological support. Tragically*,* she committed suicide months after the earthquake*,* as it was revealed that she had been suffering from severe depression during that time*.” — P12.

*“Because my husband never allows me to leave home without his permission*,* during the flood I was forced to stay in the house with my three children despite the rising water levels. With all access roads blocked*,* he couldn’t return until the next morning. As a result*,* my youngest daughter contracted a severe cold and remained ill for several weeks afterward” —* P6 from Women.

### Norms

The participants indicated that cultural norms dictate that women are responsible for tasks such as cooking, household chores, caring for disabled people, and looking after children and elderly individuals.

An expert noted, “*Women had many responsibilities. In difficult conditions*,* they had to care for children*,* cook*,* manage the cleanliness of tents and temporary shelters*,* and take care of elderly family members. Sometimes*,* women neglect their own needs and are overwhelmed by the sheer volume of tasks and responsibilities. For example*,* I saw a woman who had set up a makeshift oven under a plastic sheet. Despite all her household and childcare responsibilities*,* she had to prepare food for her family in these unsuitable conditions*,* which affected her resilience*.” — P20 from experts.

One participant shared, “*When the flood came*,* our house was destroyed. I had to take care of four children*,* one of whom was breastfeeding. I cooked on a gas stove beside the tent without proper facilities. I cleaned the tent*,* bathed the children*,* washed clothes*,* and managed countless other tasks. At night*,* I was so exhausted that I would collapse in a corner and wake up to the sound of crying. Every day was the same—constantly running around from dawn till dusk.*” — P1 from Women.

### Religious belief and practices

Participants in this study believed that belief in a non-physical world and strong faith play a significant role in enhancing the resilience of women when facing natural disasters. They reported that engaging in religious practices such as prayer and Quranic recitation helps maintain calmness and strengthens their ability to cope with the psychological pressures caused by crises.

An expert remarked, “*Belief in a nonphysical world can be extremely effective. Those who have strong faith tend to handle disasters better. For example*,* during the flood*,* we observed that women who recited prayers showed greater resilience than others*.” — P17.

A participant shared, “*Whenever I felt very down*,* I would pray two units of prayer or recite a few verses from the Quran. During those difficult times*,* these practices helped me maintain my composure. I firmly believe that these challenges are tests from God*,* and we need to rely on Him and be patient so that He may assist us*.” — P9 from Women.

## Social factors

Social factors contributing to Iranian women’s resilience in natural disasters comprised three core subcategories: (1) social interactions, (2) social support, and (3) community safety.

### Social interactions

According to the participants, the resilience of women who engage more in social activities and community involvement—such as volunteering or employment—tends to be greater. These women are also able to recover from trauma or distress more swiftly.

An expert noted, “*In our experience*,* volunteers and health workers who engage in volunteer work have greater resilience than other*s.” — P20.

A participant shared, “*We all set up tents close to our homes with family and neighbors*,* and knowing that we were not alone in this ordeal helped us maintain our morale. Whenever we had the chance*,* we would talk and spend time together*,* and we didn’t even realize how quickly time passed. We did not have the luxury to dwell on our sorrows*.” — P1 from Women.

### Social support

Study participants emphasized that women embedded in supportive family structures with robust emotional bonds exhibited significantly lower trauma exposure and demonstrated superior coping mechanisms when confronting adversity.

An expert noted, “*Consider a woman who is surrounded by a loving and supportive family. Such a family will naturally be there for her during tough times*,* and as a result*,* she will likely be more resilient than a woman whose family does not express affection or support*.” — P19.

“*Support from friends and relief groups who came to our area played a significant role in empowering me to cope with the crisis. This support provided us with a sense of comfort and prevented us from feeling isolated*.” — P6 from Women.

### Community safety

According to the study participants, violence against women increases during disasters, particularly from their partners, making women vulnerable to physical abuse, sexual assault, forced prostitution, and trafficking both within their homes and in the community.

An expert noted, “*Even in shelters*,* women often cannot ensure their safety*,* and this lack of security has a profoundly negative impact on their resilience*.” — P15.

A participant shared, *“In the initial days*,* we had no sense of security at all. Many people from other places came to the area—some to help and some to exploit the situation. The presence of so many strangers made the environment extremely unsafe*,* so we did not go alone from the tents to the bathrooms; we always gathered together for safety.”* — P8 from Women.

## Economic factors

Economic factors influencing Iranian women’s resilience to natural disasters consisted of two core subcategories: (1) assets and (2) income.

### Assets

The participants highlighted that savings, financial resources, and property ownership are crucial factors in women’s resilience during natural disasters.

One participant stated, “*I have always managed to save from selling carpets because I am skilled in weaving. During the earthquake*,* I was able to help my family*,* and we did not need assistance from anyone*.” — P3 from Women.

*“Women who owned even the smallest productive assets*,* such as small livestock herds or handicraft production tools*,* were able to recover their previous performance more quickly than others*.” — P15 from experts.

### Income

Participants noted that economically independent women faced lower risks from disasters.

“A resilience expert added: “*Financial autonomy helps women withstand disasters better and recover faster*,* especially in societies where men control resources*.” — P17 from experts.

One participant said, “*Women who had income could buy what they needed and were not dependent on their husbands to decide whether they would purchase a mobile home or not*.” — P5 from Women.

## Discussion

### Main findings

This study revealed that women possess significant characteristics and capabilities that contribute to their resilience during disasters. Similar studies have referenced comparable dimensions: individual [13], cultural [27–30], social [31–34], and economic [27, 35, 36]. In categorizing these dimensions, some articles indicate that there are similarities in the classification of dimensions across two or more categories [27, 29, 31].

Disasters reinforce, perpetuate, and exacerbate gender inequality, worsening conditions for women [37]. Women’s adaptive capacity is influenced by gender roles, which often stem from societal and cultural misconceptions [27]. Various studies [38–41] have highlighted gender discrimination and the marginalization of women as factors associated with women’s resilience. Some studies also point to limited decision-making power for women [32, 33, 40, 41] and a patriarchal culture [30, 41, 42] as factors related to gender discrimination.

Patriarchal ideologies in societies and women’s lack of access to power, structures, and resources result in male dominance in political and economic systems, increasing women’s vulnerability [27]. Empirical studies demonstrate that achieving genuine community resilience to disasters is contingent upon the full participation and active engagement of women in decision-making and leadership processes, as their unique perspectives and experiences are indispensable for effective disaster planning and response [43]. Many international strategies and frameworks also identify women as key stakeholders in disaster management [30]. However, despite the emphasis on their role in decision-making, especially in disaster risk governance, women’s opportunities for participation and decision-making remain significantly limited compared with those of men [44]. In traditional communities, women’s social interactions are often restricted compared with those of men and typically require permission from their husbands or guardians. This underscores the importance of considering social norms and cultural practices.

According to this study, educated women demonstrate better resilience. Similar studies have shown that during floods, women with higher education levels are less likely to be trapped in water or injured. For example, it is estimated that a girl who has completed high school is significantly less likely to be trapped or harmed in water than a girl who has completed only elementary school [45]. In line with the results of this study, numerous other studies [28, 29, 46, 47] have emphasized the important role of literacy and education levels in women’s resilience during disasters.

In the study by Mızrak and colleagues, data analysis The participants indicated that cultural norms dictate that women are responsible for tasks such as cooking, household chores, caring for disabled people, and looking after children and elderly individuals of 182 countries revealed that the duration of mandatory education was the only variable that significantly predicted vulnerability to disasters [48]. Educated women are aware of their rights, have greater autonomy and decision-making power, better access to job opportunities, financial independence, and social activities, and consequently, they exhibit greater resilience. These findings clearly demonstrate the crucial role of education in reducing women’s vulnerability to disasters. In fact, education not only enhances individual awareness and skills, but also enables women to transcend traditional frameworks and restrictive cultural norms, allowing them to play a more active role in society. Furthermore, higher levels of education among women can lead to shifts in social attitudes and a reassessment of gender-based division of labor, which is a key factor in strengthening the resilience of households and communities. Therefore, investing in the education of girls and women should be considered a fundamental strategy in disaster risk reduction and sustainable development programs.

Aging stands out among these factors as a particularly difficult issue. Due to their physical limitations and dependencies, elderly populations exhibit heightened susceptibility to disasters. Considering that an elderly person often feels weakened due to chronic illnesses, impaired cognitive abilities, and reduced sensory awareness, the impact of a natural disaster may be far greater [49].

With respect to personality traits, other studies, similar to the findings of this study, have pointed to traits such as optimism and humor [50, 51] as factors that enhance resilience. In a study by Semmel and colleagues, humor, whether used for distraction or building connections, was found to be helpful when facing challenges. Not taking problems too seriously reduces stress and psychological pressure during disasters, which in turn can increase resilience [52]. Women who are sensitive, irritable, or aggressive struggle to control their emotions and make appropriate decisions in critical moments, which reduces their ability to be resilient in disasters. On the other hand, women who are humorous and patient tend to manage situations better and display greater resilience during such events.

Religious beliefs and practices also increase patience and hope, helping individuals cope with difficult conditions after disasters. These findings align with those of other studies [13, 29, 39]. Relying on prayers has been highlighted as a factor in women’s resilience during natural disasters [39]. Spiritual beliefs enable individuals to trust in greater power, fostering hope [53]. Some women who endured the destruction caused by disasters-maintained peace and hope by regularly performing religious duties, such as reading the Quran and praying [54]. Similar to the results of this study, Wilkinson and colleagues suggested that the religious system of a society can contribute to resilience during disasters [55]. Other studies have shown that spirituality aids in recovery and resilience after crises and disasters [56], guides individuals to seek meaning [57, 58], and is a crucial factor in effectively dealing with negative situations [59]. This is because some individuals may experience a sense of closeness to God, leading to enhanced meaning, life purpose, and spiritual well-being [60].Religious beliefs not only provide a source of hope and comfort during crises but also enhance individuals’ sense of meaning and purpose in life. Reliance on religious rituals and prayer as effective coping strategies can contribute to stress reduction and the improvement of mental health. Therefore, incorporating spiritual and religious dimensions into post-disaster support programs is of paramount importance and can play a crucial role in fostering resilience. This comprehensive approach helps individuals better cope with the aftermath of disasters and accelerates the recovery process.

Cultural norms are integral elements of resilience, which is why they have been highlighted in studies on gender and resilience [27–30]. In most developing countries, the family serves as a strong social safety net, with responsibilities such as elder care being a cultural norm. Cultural factors that influence women’s resilience in natural disasters include defined responsibilities and duties for women in communities [13, 61], established household roles such as family and child care [39, 62], restrictive social norms, traditional gender roles [62], and the workload burden on women [63].

According to a social survey conducted among 6,000 families, mothers are two to four times more responsible and involved than fathers in their children’s education, health, and discipline [64]. Ariyabandu stated that during waves, women were taking care of their children and had to hold onto them to save them, which prevented them from protecting themselves [46]. The overwhelming volume of these responsibilities, which often intensifies during disasters due to unique conditions, significantly impacts women’s resilience.

Similar to the findings of this study, Rahman and colleagues asserted that while all women are affected by disasters, poor women are particularly impacted by the environmental destruction caused by climate change and natural disasters [37]. Access to financial resources, savings, assets, and bank accounts helps families protect themselves against the effects of natural disasters, but there is a significant gender gap in banking. The use of savings is a common coping mechanism for recovery after disasters. In Ghana, after the devastating flood of 2015, 43% of affected households relied on their savings as the primary means of coping with the impacts of the disaster [65].

According to the FinDex database, there are statistically significant differences between men and women across all aspects related to financial inclusion in 144 countries worldwide [66]. Owing to their lack of access to bank accounts, The participants indicated that cultural norms dictate that women are responsible for tasks such as cooking, household chores, caring for disabled people, and looking after children and elderly individuals tend to keep a larger portion of their assets in tangible forms, making them more vulnerable to losing their assets in disasters, which impacts their resilience [30]. Statistically, women in low-income neighborhoods experience greater impacts and slower recovery.

Various studies have pointed to factors affecting women’s resilience, including unemployment, lack of reliable monthly income, economic status [27–29, 36, 39, 41], livelihood diversification [47], and the unequal distribution of economic resources [67]. Other factors include economic dependency and poverty [33], a lack of savings, poverty, a lack of livestock ownership [35, 63], a lack of land ownership [47, 63] and a lack of assets [30, 63]. Gendered asset distribution [34] has also been highlighted as a factor that impacts women’s resilience. Additionally, an analysis of demographic data from 18 countries in South Asia, the Middle East, and Sub-Saharan Africa in 2024 indicated that women who own assets are 14% more likely to be resilient than those who do not own any assets [68]. While fundamental economic determinants—such as asset ownership and resource access—are critical to women’s capacity to withstand crises, the elimination of structural barriers and the advancement of equitable access to resources empower women to adapt more effectively during disasters. This underscores the necessity for policies and social norms that address gender-based discrimination and actively promote fair resource allocation. Such an integrated approach is essential for reinforcing women’s adaptive and recovery capabilities in the context of natural hazards.

One of the most challenging findings regarding resilience during disasters, as identified in this study, is that financial capability acts as a paradox. In cases where financial resources were tied to assets that were lost during disasters, it was viewed as a weakness. Conversely, in situations where financial resources were unaffected by disasters, it was seen as a strength.

During disasters, due to emergency conditions, meeting essential needs such as food, clothing, and shelter takes priority, and many nonessential needs are marginalized. According to the researchers of this study, income can be categorized into two groups: vulnerable to disasters and not vulnerable to disasters. Cases where women’s income is interrupted or reduced after disasters, such as jobs related to nonessential needs, negatively affect resilience. Conversely, if income is independent of disasters—such as receiving a fixed salary from the government—or if it involves resources that are more critically needed during disasters, such as jobs related to fulfilling basic needs, it positively impacts women’s resilience.

### Recommendations for future research

It is suggested that future research employ comparable qualitative methodologies across diverse cultural settings to examine the overlaps and differences in the components of women’s resilience when facing natural disasters.

### Strengths and limitations

This study, despite its valuable contributions, has several limitations that warrant consideration. First, the number of participants was limited due to the qualitative nature of the research and the difficulty in accessing experts and women affected by natural disasters. The research team endeavored to achieve conceptual saturation by purposively selecting experienced participants, particularly when participation was constrained by work commitments or accessibility issues. Additionally, due to time and resource constraints, it was not possible to cover all affected provinces. Furthermore, one woman declined to participate due to emotional distress triggered by recalling the traumatic loss of her family, which may have resulted in the omission of some important and divergent perspectives.

At the same time, this study possesses notable strengths that distinguish it from another research. For the first time in Iran, women’s resilience in the face of natural disasters has been examined through in-depth and specialized interviews with two key groups: women with disaster experience and prominent national and local experts. The qualitative approach employed enabled a deep and multidimensional understanding of women’s lived experiences and facilitated the precise identification of resilience components. Moreover, the research team’s efforts to reach data saturation and incorporate insights from national experts significantly enhanced the credibility and richness of the findings.

## Conclusions

This study provides foundational information on the dimensions and factors of women’s resilience during natural disasters, and researchers can utilize these findings to conduct further studies aimed at enhancing women’s resilience. To improve women’s resilience in natural disasters, it is necessary to address gender discrimination in broader policies and planning, such as ensuring equitable access for women to education, property ownership, employment, and managerial positions. It is essential to fundamentally consider the factors contributing to discrimination across various cultural, social, and economic dimensions.

We recommend that disaster management policies specifically include components that ensure the protection of women’s legal rights in different communities. Given that resilience is highly dependent on cultural context, the components identified in this research are applicable to Iranian society. Therefore, other communities may exhibit different components.

## Data Availability

The data sets used and/or analyzed during the current study are available from the corresponding author upon reasonable request.

## References

[1] Centre for Research on the Epidemiology of Disasters (CRED). The international disaster database. In.: Université Catholique de Louvain; 2017.

[2] Khatun H, Kabir H. Evaluation and vulnerability in disaster prone areas in Bangladesh. In: *Local Wisdom Matters.* edn. Edited by Zaveri S. Dhaka: Disaster Research Training and Management Centre (DRTMC), University of Dhaka; 2019: 116.

[3] United Nations Women. World survey on the role of women in development 2014: gender equality and sustainable development. New York: UN Women; 2014.

[4] Bradshaw S, Fordham M. Double disaster: disaster through a gender lens. Hazards, risks, and disasters in society. edn.: Elsevier; 2015. pp. 233–51.

[5] United Nations Women. Women hold key to addressing disaster risks. In. New York; 2016.

[6] Bondesson S. Why gender does not stick: exploring conceptual logics in global disaster risk reduction policy. Climate hazards, disasters, and gender ramifications. edn.: Routledge; 2019. pp. 48–66.

[7] Schipper ELF, Langston L. A comparative overview of resilience measurement frameworks. *Analyzing Indicators and Approaches; Overseas Development Institute: London, UK* 2015, 422.

[8] Talubo JP, Morse S, Saroj D. Whose resilience matters? A socio-ecological systems approach to defining and assessing disaster resilience for small islands. Environmental Challenges. 2022;7:100511.

[9] Bello O, Bustamante A, Pizarro P. Planning for disaster risk reduction within the framework of the 2030 Agenda for Sustainable Development. 2021.

[10] Girardet LH. UNDRR initial COVID-19 engagement strategy. In.: United Nations Office for Disaster Risk Reduction (UNDRR); 2020.

[11] Boström E. Are we keeping everyone safe from disasters? A study on social vulnerability and disaster risk reduction policy in Sweden. *Master’s thesis.* Lund University; 2018.

[12] Ungar M. The social ecology of resilience: A handbook of theory and practice. Springer Science & Business Media; 2011.

[13] Pourksamaei M, Pouryousefi H, Khademian A. Resilience model of rural women after the earthquake in Sarpol-e Zahab, Iran. Geographical Researches. 2021;36(4):429–36.

[14] Bahmanjanbeh F, Kohan S. A qualitative study of women’s gender roles in earthquake. J Qualitative Res Health Sci. 2017;6(3):329–40.

[15] Neher F, Miola A. Culture and resilience. In: *JRC Technical Reports EUR 28271 EN;* 2016. 2016.

[16] Smyth I, Sweetman C. Introduction: gender and resilience. Gend Dev. 2015;23(3):405–14.

[17] Bengtsson M. How to plan and perform a qualitative study using content analysis. NursingPlus Open. 2016;2:8–14.

[18] Mwita K. Factors influencing data saturation in qualitative studies. International Journal of Research in Business and Social Science (2147–4478). 2022;11(4):414–20.

[19] Ghomian Z, Yousefian S. Natural disasters in the Middle-East and North Africa with a focus on Iran: 1900 to 2015. Health Emergencies Disasters Q. 2017;2(2):53–62.

[20] Patton MQ. Qualitative research & evaluation methods. 3rd ed. Sage; 2002.

[21] Skelton T. Children, young people, UNICEF and participation. Global childhoods. edn.: Routledge; 2013. pp. 165–81.

[22] Barriball KL, While A. Collecting data using a semi-structured interview: a discussion paper. J Adv Nurs. 1994;19(2):328–35. 8188965 10.1111/j.1365-2648.1994.tb01088.x

[23] Saunders B, Sim J, Kingstone T, Baker S, Waterfield J, Bartlam B, et al. Saturation in qualitative research: exploring its conceptualization and operationalization. Qual Quant. 2018;52:1893–907. 29937585 10.1007/s11135-017-0574-8 PMC5993836

[24] Creswell JW, Poth CN. Qualitative inquiry and research design: choosing among five approaches. Sage; 2016.

[25] Lindgren B-M, Lundman B, Graneheim UH. Abstraction and interpretation during the qualitative content analysis process. Int J Nurs Stud. 2020;108:103632. 32505813 10.1016/j.ijnurstu.2020.103632

[26] Schwandt TA, Lincoln YS, Guba EG. Judging interpretations: but is it rigorous? Trustworthiness and authenticity in naturalistic evaluation. New Dir Eval. 2007. 10.1002/ev.223.

[27] Enarson E, Chakrabarti P. Women, gender and disaster: global issues and initiatives. Women, gender and disaster: global issues and initiatives. xviii; 2009. pp. 380–xviii.

[28] Pongponrat K, Ishii K. Social vulnerability of marginalized people in times of disaster: case of Thai women in Japan tsunami 2011. Int J Disaster Risk Reduct. 2018;27:133–41.

[29] Shooshtari S, Abedi MR, Bahrami M, Samouei R. Determining the reasons behind women’s vulnerability during disasters: a qualitative study. Saf Promotion Injury Prev (Tehran). 2017;5(1):51–8.

[30] Erman A, De Vries Robbe SA, Thies SF, Kabir K, Maruo M. Gender dimensions of disaster risk and resilience: Existing evidence. In: World Bank Policy Research Working Paper. 2021.

[31] Alam K, Rahman MH. Women in natural disasters: a case study from Southern coastal region of Bangladesh. Int J Disaster Risk Reduct. 2014;8:68–82.

[32] Azad AK, Hossain KM, Nasreen M. Flood-induced vulnerabilities and problems encountered by women in Northern Bangladesh. Int J Disaster Risk Sci. 2013;4:190–9.

[33] Singh D. Gender relations, urban flooding, and the lived experiences of women in informal urban spaces. Asian J Womens Stud. 2020;26(3):326–46.

[34] Segnestam L. Gendered experiences of adaptation to drought: patterns of change in El Sauce, Nicaragua. Latin Am Res Rev. 2017;52(5):807–23.

[35] Naz F, Saqib SE. Gender-based differences in flood vulnerability among men and women in the Char farming households of Bangladesh. Nat Hazards. 2021;106(1):655–77.

[36] Ostovar Izadkhah Y. Women and natural disasters: vulnerable in accidents or capable of managing the crisis. J Rescue Relief. 2010;2(2):71–80.

[37] Rahman MS. Climate change, disaster and gender vulnerability: a study on two divisions of Bangladesh. Am J Hum Ecol. 2013;2(2):72–82.

[38] Detraz N, Peksen D. In the aftermath of Earth, Wind, and Fire: natural disasters and respect for women’s rights. Hum Rights Rev. 2017;18:151–70.

[39] Ajibade I, McBean G, Bezner-Kerr R. Urban flooding in Lagos, Nigeria: patterns of vulnerability and resilience among women. Glob Environ Change. 2013;23(6):1714–25.

[40] Phan LT, Jou SC, Lin J-H. Gender inequality and adaptive capacity: the role of social capital on the impacts of climate change in Vietnam. Sustainability. 2019;11(5):1257.

[41] Yumarni T, Amaratunga D, Haigh R. Assessing gender vulnerability within post-earthquake reconstruction: case study from Indonesia. Procedia Econ Finance. 2014;18:763–71.

[42] Ashraf MA, Secretariat B. Gender issues in disaster: understanding the relationships of vulnerability, preparedness and capacity. Environ Ecol Res. 2015;3:136–42.

[43] Alam K, Rahman MH. The Role of Women in Disaster Resilience. In: Handbook of Disaster Risk Reduction & Management. edn.:697–719.

[44] Zaidi RZ, Fordham M. The missing half of the Sendai framework: gender and women in the implementation of global disaster risk reduction policy. Prog Disaster Sci. 2021;10:100170.

[45] Muttarak R, Pothisiri W. The role of education on disaster preparedness: case study of 2012 Indian Ocean earthquakes on Thailand’s Andaman Coast. Ecol Soc. 2013. 10.5751/ES-06101-180451.

[46] Ariyabandu MM. Sex, gender and gender relations in disasters. Women Gend Disaster: Global Issues Initiatives. 2009:5–17.

[47] Ncube A, Mangwaya PT, Ogundeji AA. Assessing vulnerability and coping capacities of rural women to drought: a case study of Zvishavane district, Zimbabwe. Int J Disaster Risk Reduct. 2018;28:69–79.

[48] Mızrak S, Çam H. Determining the factors affecting the disaster resilience of countries by geographical weighted regression. Int J Disaster Risk Reduct. 2022;81:103311.

[49] Pekovic V, Seff L, Rothman MB. Planning for and responding to special needs of elders in natural disasters. Generations: J Am Soc Aging. 2007;31(4):37–41.

[50] Ferrer R, Lagos D. Women’s capacities in adversities: categorizing women’s disaster resilience through exploratory factor analysis. J Hum Ecol. 2016;5(1):1–11.

[51] Garrison MB, Sasser DD. Families and disasters: Making meaning out of adversity. Lifespan perspectives on natural disasters: Coping with Katrina, Rita, and other storms. 2009:113–130.

[52] Semmel S. Things are Going to Get a Lot Worse Before They Get Worse: Humor in the Face of Disaster, Politics, and Pain. In: The Popular Culture Association National Conference. Philadelphia, PA, USA; 2020.

[53] Bocacao HR. Stories women tell: exploring lived experiences with natural disasters in the coastal and upland communities of Sagñay, Camarines Sur. Ho Chi Minh City Open Univ J Sci. 2023;13(1):117–28.

[54] Sohrabizadeh S, Jahangiri K, Khani Jazani R. Religiosity, gender, and natural disasters: a qualitative study of disaster-stricken regions in Iran. J Relig Health. 2018;57:807–20. 28425006 10.1007/s10943-017-0398-9

[55] Wilkinson O. Faith and Resilience After Disaster. The Case of Typhoon Haiyan Olivia Wilkinson Report Commissioned Misean Cara. 2015.

[56] Ikizer G, Karanci AN, Doğulu C. Exploring factors associated with psychological resilience among earthquake survivors from Turkey. J Loss Trauma. 2016;21(5):384–98.

[57] Parida PK. Natural disaster and women’s mental health. Soc Change. 2015;45(2):256–75.

[58] Brielle G, Delano A, Grissham G. Contextual theology and the role of religion in post-disaster recovery: a theological and humanitarian perspective. Ministries Theol. 2024;2(1):18–26.

[59] Blanc J, Rahill GJ, Laconi S, Mouchenik Y. Religious beliefs, PTSD, depression and resilience in survivors of the 2010 Haiti earthquake. J Affect Disord. 2016;190:697–703. 26600411 10.1016/j.jad.2015.10.046

[60] Milstein G. Disasters, psychological traumas, and religions: resiliencies examined. Psychol Trauma Theory Res Pract Policy. 2019;11(6):559. 10.1037/tra0000510 31478722

[61] Kulatunga U, Wedawatta G, Amaratunga D, Haigh R. Evaluation of vulnerability factors for cyclones: the case of Patuakhali, Bangladesh. Int J Disaster Risk Reduct. 2014;9:204–11.

[62] Chowdhury JR, Parida Y, Goel PA. Does inequality-adjusted human development reduce the impact of natural disasters? A gendered perspective. World Dev. 2021;141:105394.

[63] Maltitz LV, Bahta YT. Empowerment of smallholder female livestock farmers and its potential impacts to their resilience to agricultural drought. AIMS Agric Food. 2021;6(2):603–30.

[64] Douki S. Work and Women’s Mental Health. Contemporary Topics in Women’s Mental Health: Global perspectives in a changing society. 2009:423–42.

[65] Erman AE, Tariverdi M, Obolensky MAB, Chen X, Vincent RC, Malgioglio S, Maruyama Rentschler JE, Hallegatte S, Yoshida N. Wading out the storm: The role of poverty in exposure, vulnerability and resilience to floods in Dar Es Salaam. World Bank Policy Research Working Paper. 2019(8976).

[66] Antonijević M, Ljumović I, Ivanović Đ. Is there a gender gap in financial inclusion worldwide? J Women’s Entrepreneurship Educ. 2022(1–2):79–96.

[67] Castelo S, Antunes L, Ashrafuzzaman M. The impact of the climate crisis on gender inequality. Looking to the frontlines in search of priorities for policy. Front Sustain Cities. 2024;6:1304535.

[68] Amir-ud-Din R, Naz L, Ali H. Relationship between asset ownership and women’s empowerment? Evidence from DHS data from 18 developing countries. J Demogr Econ. 2024;90(2):154–75.

[69] General Assembly of the World Medical Association.World Medical Association. Declaration of helsinki: ethical principles for medical research involving human subjects. J Am Coll Dent. 2014;81(3):14–8. 25951678

